# Integrated modelling of crop production and nitrate leaching with the *Daisy* model

**DOI:** 10.1016/j.mex.2016.04.008

**Published:** 2016-04-26

**Authors:** Kiril Manevski, Christen D. Børgesen, Xiaoxin Li, Mathias N. Andersen, Per Abrahamsen, Chunsheng Hu, Søren Hansen

**Affiliations:** a Department of Agroecology, Aarhus University, Blichers Allé 20, 8830 Tjele, Denmark; b Institute of Genetics and Developmental Biology, Chinese Academy of Science, Huaizhong Lu 286, 050021 Shijiazhuang, China; c Sino-Danish Center for Education and Research, Zhongguancun College, 271 N 4th Ring Road, Haidian, 100080 Beijing, China; d Department of Plant and Environmental Sciences, University of Copenhagen, Thorvaldsensvej 40, Frederiksberg, 1871 Copenhagen, Denmark

**Keywords:** Integrated modelling of crop production and nitrate leaching at field scale, Calibration, Sensitivity analysis, Validation, Re-validation, Model application

## Abstract

An integrated modelling strategy was designed and applied to the Soil-Vegetation-Atmosphere Transfer model *Daisy* for simulation of crop production and nitrate leaching under pedo-climatic and agronomic environment different than that of model original parameterisation. The points of significance and caution in the strategy are:

• Model preparation should include field data in detail due to the high complexity of the soil and the crop processes simulated with process-based model, and should reflect the study objectives. Inclusion of interactions between parameters in a sensitivity analysis results in better account for impacts on outputs of measured variables.
• Model evaluation on several independent data sets increases robustness, at least on coarser time scales such as month or year. It produces a valuable platform for adaptation of the model to new crops or for the improvement of the existing parameters set. On daily time scale, validation for highly dynamic variables such as soil water transport remains challenging.

• Model application is demonstrated with relevance for scientists and regional managers. The integrated modelling strategy is applicable for other process-based models similar to *Daisy*. It is envisaged that the strategy establishes model capability as a useful research/decision-making, and it increases knowledge transferability, reproducibility and traceability.

## Graphical abstract

**Figure:**
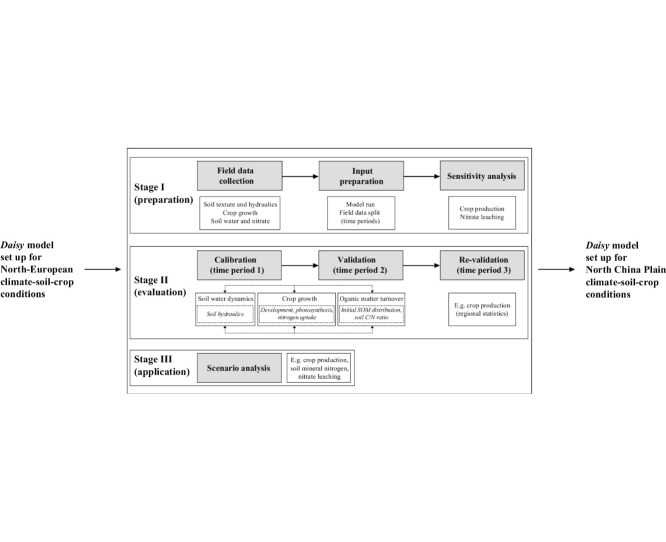

## Method details

**Fig. 1:**
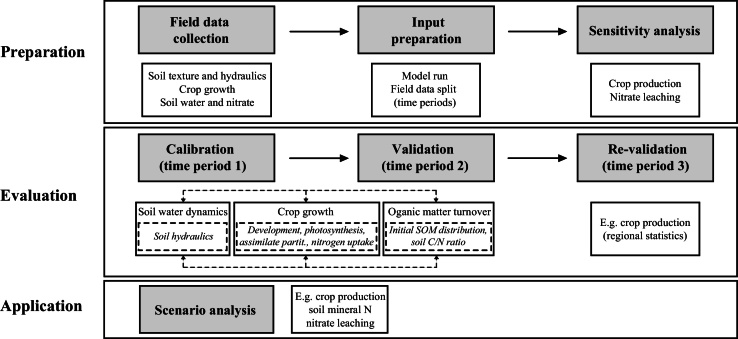
Main stages of the integrated modelling strategy to set up the *Daisy* model for the climate-crop-soil conditions in the North China Plain. Grey boxes denote main steps; white boxes denote main variables/processes; dashed-line boxes denote parameter sets; dash-arrows denote iterations. SOM is soil organic matter; C/N ratio is the carbon-nitrogen ratio.

The one-dimensional Soil-Vegetation-Atmosphere Transfer (SVAT) model *Daisy* [8] was primarily designed and parameterised with measurements from sandy soils and single crop rotations under the sub-humid temperate climate of Northern Europe. Calibration of the model for different pedo-climatic and agronomic environments is not straight-forward due to its field-scale nature and process-based structure. An integrated modelling strategy (Fig. 1) was designed and applied to *Daisy* for simulation of crop production and nitrate leaching in maize—winter wheat double crop rotation on silty loam soils in semi-arid China. The integrated modelling strategy is applicable for other process-based models similar to *Daisy*.

### Model preparation

#### Field data collection

Field data collection reflects the study objective, in this case calibration of a process-based model for crop growth and soil nitrogen (N) processes. Field measurements were conducted at Luancheng Agro-Ecosystem Experimental Station (37°53′15″N, 114°40′47″E), the Chinese Academy of Science, Shijiazhuang, the North China Plain (NCP). The field measurements were performed on maize and winter wheat grown in a double crop rotation, i.e., maize was typically sown in June and harvested in October, followed by winter wheat that was harvested the following June. Complete block N response plots with three replicates were established in 1998 on two neighbouring fields: A (17% topsoil clay, 30% subsoil clay, straw incorporated after each crop harvest) and B (5% topsoil clay, 14% subsoil clay, straw removed from the field after each crop harvest). The soil texture, organic matter and bulk density were described by Wang et al. [19] for field A and [10] for field B. The plots at both fields were top dressed with urea fertiliser at annual rates of 0, 200, 400 and 600 kg N ha^−1^, referred to hereafter as N0, N200, N400, and N600, respectively. Half of the fertiliser amount was applied on maize during flowering in August (field A) or at sowing (field B), while the other half was applied on winter wheat either in split dosage before sowing in October and at stem elongation in April (field A) or at sowing (field B). The field data covered the period June 2007–June 2013 (12 crop seasons) for field A, and June 2001–June 2004 (6 crop seasons) for field B (Table 1). Recent crop measurements were conducted in detail from June 2012 to June 2013 at field A and included leaf area index (LiCor3100, Li-Cor Environmental, USA), dry matter (DM; oven-drying) and N content (Kjeldahl System 2300, Tecator, Sweden) of crops organs (leaf, stem, grain) at juvenile, flowering and harvest. In addition, by digging two soil pits and exposing the soil profiles from 0 to 200 cm depth, maize roots were sampled in October 2013 according to Ahmadi et al. [2]. Maize root length density (RLD) and maximum rooting depth were determined according to Tennat [17], whereas winter wheat RLD and maximum rooting depth were adopted from Zhang et al. [20].

Actual evapotranspiration (*AET*) was obtained with a weighing lysimeter (3 m^2^, 2.5 m depth, 0.02 mm resolution of water loss) installed 100 m from the fields and managed similarly in terms of crop, irrigation and fertilisation; for details and data, see Li et al. [10]. The *AET* was used to calculate the drainage (*D*) according to the water balance method [14], assuming no surface runoff and negligible upward water movement at the site:

(1) $$D_{z}=P+I+\Delta SW_{z}-AET$$

where *z* is soil depth, *P* is precipitation, *I* is irrigation, *ΔSW* is change in soil water storage (24 h change in water content measured with neutron probes) and *AET* is actual evapotranspiration. Since the ammonium concentrations were negligible, leaching was obtained by multiplying the estimated drainage by the measured nitrate concentrations:

(2) $$N_{leach}=\sum^{,}D_{z,i}\cdot c_{i}$$

where *i* is time of measurement, *c* is measured nitrate concentration, and *N_leach_* is nitrate leaching.

Daily management practices such as time of planting and harvest of the crops, time and rate of N fertiliser and irrigation application, tilling date and depth, and fraction of crop straw returned to the soil, were all recorded (Table 2).

#### Input preparation

Input files necessary to run *Daisy* include daily weather, soil, crop and management information.

- Weather: daily air temperature, precipitation, solar radiation, wind speed and relative humidity were recorded at Luancheng station. The respective values for wet and dry deposition of 7.5 ppm and 10 kg N ha^−1^ for ammonium and 2.5 ppm and 5 kg N ha^−1^ for nitrate required by the *Daisy* weather file were approximated from Zhang et al. [21]. However, modelers should use measured values when these are available in order to increase the accuracy of the simulated atmospheric N deposition.
- Soil: two soil files, one for each field, were constructed with the measured soil properties. The van Genuchten-Mualem soil hydraulic parameters, describing soil water retention and unsaturated hydraulic conductivity at different soil water pressure potentials, were optimised against measured water retention in field A in 2007 (UMS GmbH, Germany, unpublished) with the nonlinear least square algorithm of RETC code [18]; the parameters were used for both fields and were given as input to *Daisy*. Further, for both soils the column was discretised with 2 cm increment from 0 to 60 cm, 5 cm increment from 60 to 170 cm and 10 cm increments from 170 to 210 cm.
- Crop: “Pioneer maize” and “Winter wheat” were selected as respective maize and winter wheat input files because these initially provided simulation results close to the measured DM dynamics compared with the other parameterisations in the *Daisy* library that are based on specific experiments in Denmark.
- Management information: the daily management practices at the two fields recorded during field data collection were used to construct the management file.

#### Sensitivity analysis

**Fig. 2:**
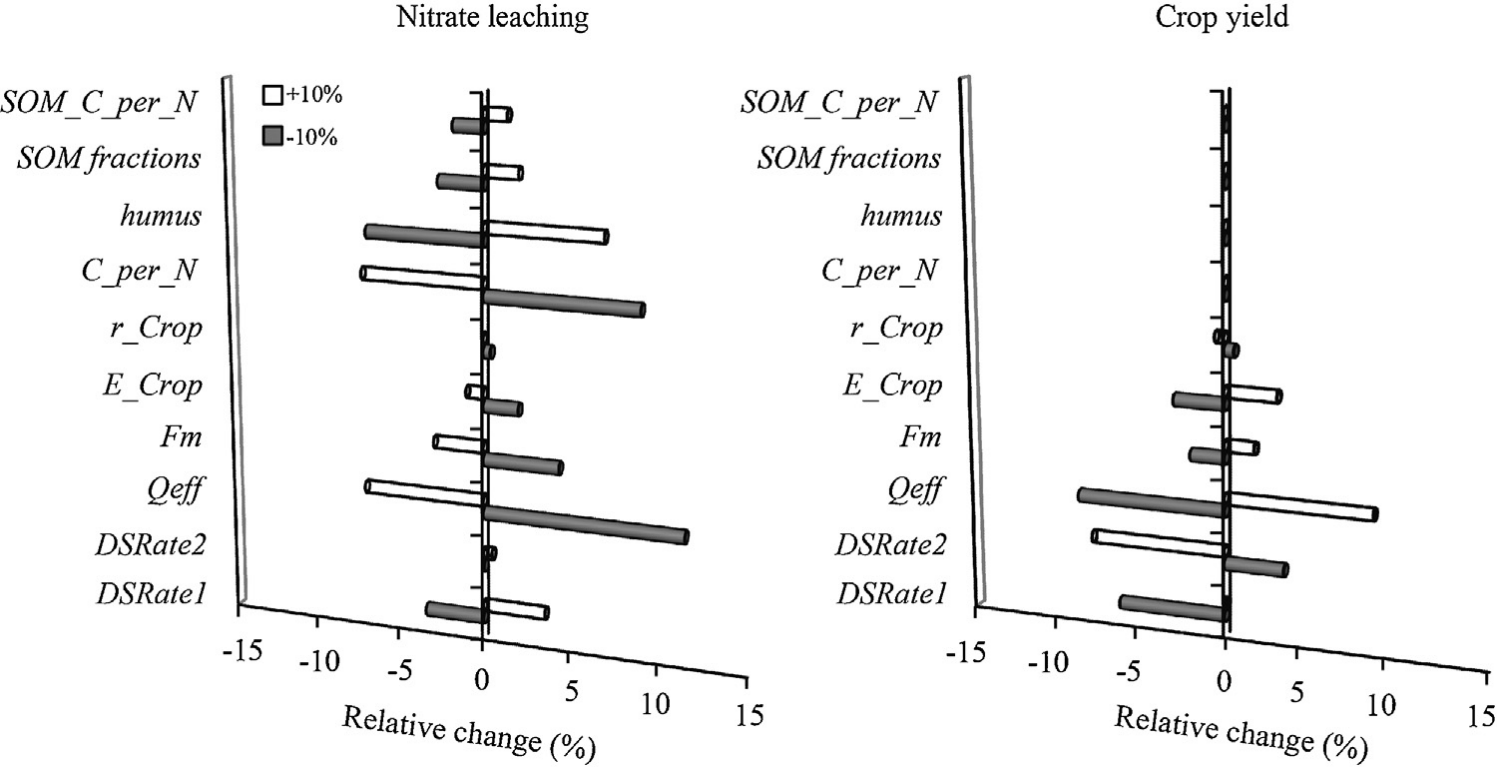
Relative sensitivity of soil and crop parameters on simulated nitrate leaching (kg N ha^−1^) and crop yield (Mg ha^−1^) with the *Daisy* model. For convenience, the changes of conversion efficiencies *(E_Leaf*, *E_Stem* and *E_SOrg*) and respiration coefficients (*r_Leaf*, *r_Stem*, *r_SOrg*) are represented by one respective working parameter (*E_Crop* and *r_Crop*), which is the average change of the three parameters. Description of all parameters is given in Table 4, Table 5.

Performing a model sensitivity analysis for all parameters would be a monumental task, therefore the choice of parameters depend on the study objective. Expert knowledge and model manuals are sound ways to select parameters. Key parameters directly related to crop development, leaf photosynthesis and net mineralisation (topsoil) were tested for sensitivity by the method of mono-factor analysis in order to help finding sensitive parameters that must be focused on when calibrating crop yield and nitrate leaching. The *Daisy* model was run for N400 treatment in field A with the actual climate 2000–2013, soil and management. The sensitivity tests were conducted by systematically increasing and decreasing a single parameter value with 10% while keeping all other input parameters constant as in the baseline scenario in order to diagnose the response of the crop yields and nitrate leaching (Fig. 2). The interacting effects between parameters were accounted for by testing the sensitivity of the crop yields to change in selected soil parameters, and that of nitrate leaching by changing the crop parameters. The low sensitivity of crop production on change in the tested soil parameters is due to sufficient nitrate in the soil column for the treatments (except N0); users should include more nitrate- (or soil water-) limited treatments, when available, to obtain deeper insight into the change of model outputs.

### Model evaluation

For all simulations described next: 1. daily precipitation, irrigation and potential evapotranspiration were set as the atmospheric upper boundary and deep groundwater was the lower boundary, 2. all simulations started on 15 June 1998, and run until 15 June 2013 for field A and 15 June 2004 for field B, and 3. visual performance analysis and four objective measures were used to evaluate model goodness of fit to the measured data i.e. root mean squared residuals (*RMSR*), mean absolute deviation (*Dev*), Nash-Sutcliffe model efficiency (*ME*), and coefficient of determination (*R^2^*).

#### Calibration

The field data were split up according to the “hold out” method [3] and those used for calibration included the highest volume of details (Table 1). Although the method of splitting the data for calibration and validation affects the bias of the estimated parameter value, the calibration data needs to be the most detailed. The model was calibrated by ‘trial and error’, first by fitting the soil water dynamics, then the crop growth and N uptake patterns, and last the soil nitrate dynamics, with an iteration process in between (Fig. 1).

**Fig. 3:**
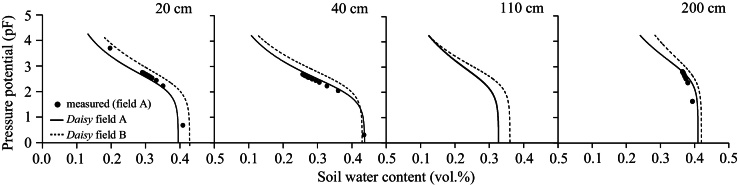
Soil water retention curves used in *Daisy* to simulate maize and winter wheat fields at Luancheng station, the North China Plain. pF = log_10_ (cm H_2_O). The depths correspond to the soil horizons used in the model; no measured data at 110 cm depth.

**Fig. 4:**
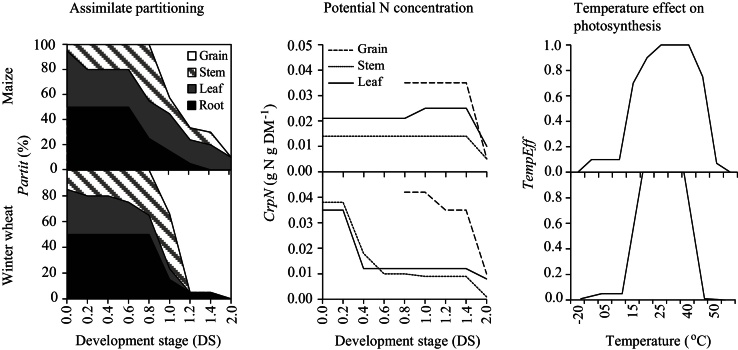
Crop parameters used in *Daisy* to simulate maize and winter wheat fields at Luancheng station, the North China Plain.

- From the already optimised soil hydraulic parameters, calibration was made on saturated hydraulic conductivity (*Ksat*, matching point in the Mualem equation) and on the *l*-parameter until the simulated soil water contents at the two fields approached the measured ones. At this stage, simulated soil water drainage and *AE*T were also followed and matched to their field values as these processes largely depend on the soil hydraulics. The final values of the soil water retention and hydraulic parameters are presented in Table 3 and Fig. 3.
- For the crop calibration, three main steps were performed: 1. a first set of parameters for phenology, canopy development, partitioning of DM between crop organs and their N concentrations was parameterised based on the field measurements; 2. a second set of parameters detected by the sensitivity analysis and related to photosynthesis and assimilate production was calibrated by iterative change until the dynamics of the variables (crops DM and N content) were simulated with the highest accuracy; 3. sensitive parameters from the above steps were fine-tuned to improve the simulation of the crop growth patterns; the aim was to derive a final set of parameters able to simulate maize and winter wheat in both fields in all calibration years (Table 4, Fig. 4). It is emphasised that the crop parameters *DSRate1* and *DSRate2* are among the first to be calibrated to measured dates of flowering and maturity because many variables in *Daisy* are in function of crop development, and their calibration exerts noticeable influence on the simulation of both crop yield and nitrate leaching (Fig. 2).
- The calibration of the nitrate leaching was done by altering the soil organic matter (SOM) turnover compartment. Historical period of five years before the experiment year (a “warm-up” period) with known data for N input from fertiliser and crop residues was included for each simulation in order to approximate the annual net mineralisation i.e. the release of N from mineralisation of the organic matter pools in the model [4]. The net mineralisation is sensitive to the distribution of SOM at the start of the simulation period, hence the model was initialised with *SOM_fractions*, a sensitive parameter (Fig. 2) reflecting the amount of SOM in the slow, fast and inert pools of the model. This parameter was first calibrated for the N0 treatment until simulated crops N at harvest matched the measured values. An inherent assumption was that the water balance is correct; otherwise the nitrate leaching will also be an unknown. The ratio was then used and adjusted, if needed, to simulate the other treatments. The ammonia volatilisation was simulated assuming that increased temperature, soil wetness before application and amount of urea increases soil pH and thus lead to relatively higher fertiliser losses [12]. Hence, losses from 50, 100, 150, 200, and 300 kg N ha^−1^ fertiliser amounts were set at 5%, 10%, 12%, 15%, and 17%, respectively. The parameters relevant for the organic matter turnover are given in Table 5.

The statistics of the model calibration were acceptable (Table 6). Given the iterations in the integrated modelling framework, the calibration contributes decisively towards obtaining a better understanding of the model structure and its correspondence to the real world system, although it is inherently subjective and can be time-consuming. A simulated 10-year period takes about 3 min in real time on standard computers, and users may implement semi-automated procedures for parts of the calibration scheme, taking into account realistic values of the biophysical parameters present in the model.

#### Validation

The model was validated on the rest of the field data and the statistics were acceptable (Table 7), despite a few negative *ME* values for aboveground N that probably occurred because of the very small variability in the measured yield at harvest. Hence, the newly parameterised maize and winter wheat are a significant outcome of the crop calibration and a valuable platform for future simulation studies with the *Daisy* model in China or for improvement of the existing parameters set. The relatively large discrepancy (highest *RMSR*, lowest *ME*) for the soil water contents was probably because of the problems associated with getting true values for soil water retention and hydraulics for both fields (Fig. 3). Therefore, there is a need for measuring soil water retention and hydraulic properties of the actual field to be simulated with the model in order to increase the accuracy of soil water dynamics at daily time scale. On a coarser time scale, which was the main intention in the method, the model performed well.

One of the main purposes of the integrated modelling strategy is to keep track on the simulation of all the major components of the mass balance of interest, in this case the N balance. The important N components other than N loss by nitrate leaching fell well within their measured ranges for the same or similar silty loam fields in the NCP with comparable management practices (Table 8).

#### Re-validation

**Fig. 5:**
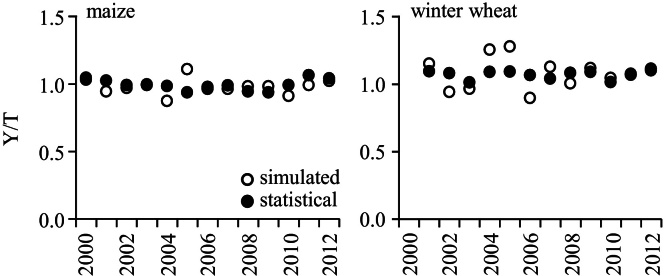
Comparison between simulated and statistical (n = 17) crop grain yield as de-trended series (Y = yield component, T = linear trend component) from 2000 to 2012 in Shijiazhuang prefecture, the North China Plain.

The *Daisy* model was re-validated for the county-level statistical crop yields (17 counties) in Shijiazhuang Prefecture from 2001 to 2012. For this long-term simulation, the soil file for field A was used as a dominant agricultural soil in the prefecture and the model was initialised with 1.3% organic matter. The statistical crop yields, as well as the N fertiliser input, were taken from the accredited China Knowledge Resource Integrated Database (http://epub.cnki.net). Crops straw was removed after harvest and the other management practices (time of sowing and harvest, time and amount of irrigation) were left as in the calibration procedure. The model performance was acceptable (Fig. 5). While these results reveal that the model provides predictability for crop yield at regional scale as well, users may re-validate the original validation effort (or certain part of it) on other biophysical variables and spatio-temporal scales, depending on the study objectives and the available data.

Some disadvantages of the integrated modelling strategy relate to the number of parameters (and variables, simulated versus measured) that increases as the modelling work progresses and that users have to account for. This may result in developing comprehensive but less robust outcome by obscuring the most influential parameters that generate the “correct” dynamics behaviour. Thus, the sensitivity analysis is an important step for “ranking” the important parameters. Also, the treatment of space is very limited because the *Daisy* model is one-dimensional, thus the number of simulations increases with increasing number of actual fields at the same site to be simulated; this can be partially compensated by using a batch file to run many simulation instances in parallel on the same computational node.

### Model application

**Fig. 6:**
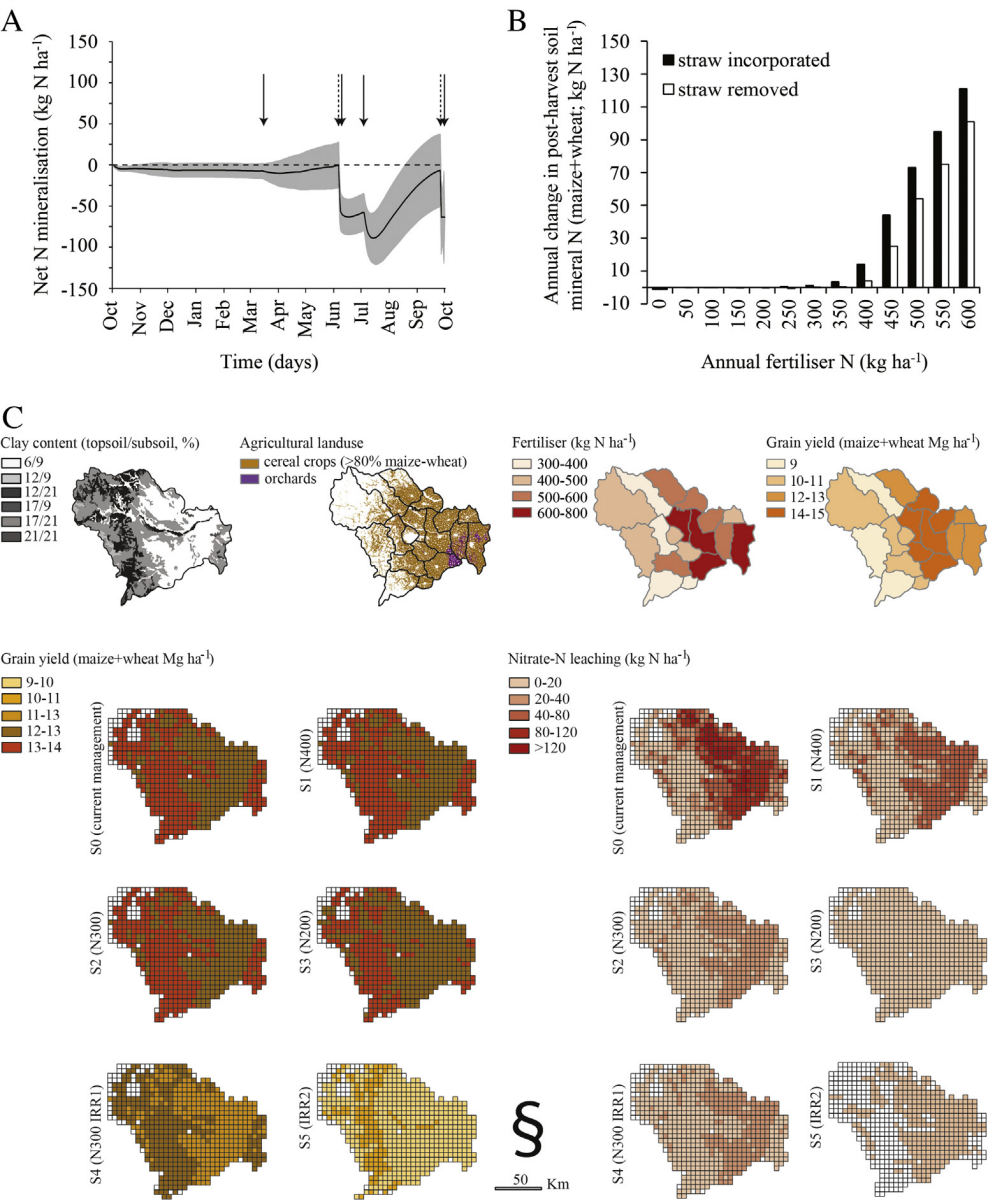
Application of the *Daisy* model setup for the North China Plain pedo-climatic conditions. A: Annual (cumulative) net N mineralization at Luancheng (silty loam soil); results are presented as three-year (2009–2011) average for the treatments, shaded areas indicate year variation (±1 standard deviation); full-arrow indicates fertiliser application, dashed-arrow indicates harvest. B: Annual change in residual soil mineral nitrogen (0–2 m soil) under different straw managements and nitrogen fertiliser rates at Luancheng (silty loam soil); each bar represents the slope from linear regression of post-harvest soil mineral nitrogen (6 October for maize, 12 June for winter wheat) over 20-year continuous weather data (1991–2012), and annual nitrogen fertiliser is sum of the crop nitrogen fertiliser (maize + winter wheat). C: Regional variation in crop yield (maize + wheat) and nitrate leaching (2 m soil depth) for current management (S0) and different scenarios (S1–S5); description of the scenarios can be found in the text.

The final stage of the integrated modelling strategy constitutes the ultimate use of the model in agro-environmental analysis as a research or a decision support tool. Two plausible model applications, at a field- and at a regional scale, are summarised and presented. In the field-scale application, the calibrated model was firstly used to investigate the organic N turnover for the studied soil, with the results (Fig. 6A) showing large immobilisation of soil mineral N with straw incorporation after harvest due to the high C/N ratio of the crop residues (Table 4); Net mineralisation dominated the soil system shortly thereafter, especially during maize season in summer when both temperature and soil moisture are high. The model was then run for field A, a dominant agricultural soil in Luancheng, for the period 1991–2013 with maize—winter wheat double crop rotation receiving annual fertiliser rates from 0 to 600 kg N ha^−1^ at 50 kg N ha^−1^ increments and split half between the crops; initialisation was made with 1.3% topsoil organic matter and crops straw was either returned to the soil after each crop harvest (straw incorporated scenario) or was completely removed from the field (straw removed scenario). The results (Fig. 6B) pointed on an annual fertiliser rate of 300 kg N ha^−1^ with straw incorporation as balance between obtaining a high crop yield and a low environmental impact i.e. accumulation of soil mineral N, whereas annual fertiliser rate of at least 350 kg N ha^−1^ should be considered when straw is removed from the field.

The *Daisy* model was also run for several soil-management combinations for the Shijiazhuang region. Based on the re-validation exercise (current management, S0), three scenarios (S1, S2 and S3) were derived and considered three annual N fertilizer rates uniformly applied across the region i.e. 400, 300 and 200 kg N ha^−1^ for S1, S2 and S3 respectively. In these simulations, the crops straw was incorporated into the soil after harvest as opposed to S0. Two additional scenarios (S4 and S5) were derived from S2 and introduced deficit irrigation. Since the time of irrigation for both crops was considered as strategic in S0, only the irrigation amount was decreased only in winter wheat (by 25%; S4) and in both winter wheat and summer maize (by 25% in winter wheat and rainfed maize; S5). Single-station climate data from Luancheng station was used to covered the region (rather low precipitation and especially temperature gradient) and soil data were taken from [16] and aggregated to six columns. Other management practices were left as in the calibration. The simulated crops yield and nitrate leaching outputs for each climate-soil-management combination were upscaled on 5 × 5 km grid using information on cropping area (Fig. 6C). The results depicted the spatial variation in crops yield and nitrate leaching in the region under the current management conditions, with comparable results between the upscaled simulated- and the statistical yield. The upscaled yield was higher than the statistical yield for the north-west (probably due to higher clay content-high soil water and N retention), but this mountainous region is of low agronomic importance with limited cropping area. The scenario results pointed on options for regional N management in relation to N fertiliser rate, straw incorporation and field irrigation that will return high crop yield and low nitrate leaching.

## Additional information

### Description of the *Daisy* model

*Daisy* (version 5.19 used in this study) is a one-dimensional SVAT model that simulates crop-, water-, carbon- (C), and N dynamics as driven by daily weather data and daily field management operations [8]. The main compartments and their process descriptions used in this study are briefly explained in the following. The soil hydrology compartment simulates water transport (Richard’s equation), heat fluxes (Fourier’s law), and reference evapotranspiration (FAO Penman-Monteith equation used in the present study). The crop compartment simulates crop development and growth. Crop development stages (DS) from emergence (DS = 0) to flowering (DS = 1), and from flowering to maturity (DS = 2) are simulated with the respective daily development rates. The leaf photosynthesis is described by a light response curve [6] modified with temperature and senescence functions. The gross photosynthesis is calculated by integration of the leaf photosynthesis over the canopy and is affected by environmental stresses in relation to water, N and development. Assimilates (a net result of photosynthesis and respiration-growth and maintenance) are allocated to leaf, stem and storage organ (grain) as a function of root/shoot ratio at various DS. Root distribution depends on root mass and, by default, decreases exponentially with soil depth according to Gerwitz and Page [5]. The SOM turnover compartment simulates mineralisation and immobilisation using three main discrete pools of C and N, i.e. SOM, added organic matter (AOM) from crop residues, rhizodeposition and organic fertilisers, and soil microbial biomass (SMB). These pools are further subdivided according to first-order kinetics into slow (indexed 1, e.g. SOM1) and fast (indexed 2, e.g. SOM2), in addition to one inert pool (SOM3) that does not contribute to the turnover. N immobilisation occurs to the pool with a C/N ratio smaller than the C/N ratio of the source pool; otherwise, organic N mineralises to ammonium-N that is nitrified to nitrate-N and dissolved in the soil water. The mineralisation of SOM1, SOM2 and SMB1 depends on soil temperature, clay and water content, while mineralisation of SMB2 and AOM depends on soil temperature and water content. Nitrification-denitrification is a function of soil temperature, ammonium and water content. The transport of soil nitrate and ammonium, and hence their leaching, is simulated by the convection-dispersion equation. Ammonia volatilisation is given as a percentage of the ammonium fertiliser amount at the time of application. Atmospheric N input to the soil is based on ammonium and nitrate concentrations in the rain, which are specified in the weather file. Further details on model equations and assumptions are available elsewhere [1], [7], [8], [13].

The *Daisy* model is freely available (http://daisy.ku.dk/).

## Figures and Tables

**Table 1:** Outline of the field experiments with a maize—winter wheat double crop rotation at Luancheng station, the North China Plain, used to set up the *Daisy* model. Nitrogen (N) fertiliser is annual, with each crop receiving half of the amount.

Field	A							B		
	Validation						Caibration	Validation	Calibration	
Year	2006–07	2007–08	2008–09	2009–10	2010–11	2011–12	2012–13	2001–02	2002–03	2003–04
Precipitation (mm)	523	596	534	366	338	414	570	327	445	525
Irrigation (mm)	274	240	240	330	390	360	295	698	420	330
Temperature ( °C)	13.2	12.6	13.0	11.8	12.4	12.5	12.1	13.2	12	12.6
N fertiliser (kg N ha^−1^)										
N0 (0)	C^a^	C	C				CnWDN			
N200 (200)	C	C	C	C	Cn	CnDN	CnWDN	CnWEDN	CnWEDN	C
N400 (400)	C	C	C	C	Cn	CnDN	CnWDN	CnWEDN	CnWEDN	C
N600 (600)	C	C	C	C	Cn	CnDN	CnWDN	CnWEDN	CnWEDN	C

a C—crop yield at harvest; n—crop nitrogen at harvest, W—daily soil water content; E—monthly evapotranspiration; D—annual soil water drainage; N—annual nitrate leaching.

**Table 2:** Management details for maize and winter wheat in the experiments on field A (straw incorporated) and field B (straw removed) at Luancheng station, the North China Plain.

Field	Year	Crop	Sowing	Harvest	Fertilisation	Irrigation	Straw at harvest	Reference
			dd mm					
A	2007	wheat	10.10	14.06	03.10; 07.04	03.10; 07.04; 19.05	80% incorporated; 20% removed (jointly with grain); 10 cm stubble left.	[20]
	2007	maize	15.06	01.10	27.07	19.06; 29.07		
	2008	wheat	10.10	14.06	03.10; 07.04	03.10; 07.04; 19.05		[15]
	2008	maize	15.06	01.10	27.07	19.06; 29.07		
	2009	wheat	05.10	10.06	05.10; 10.04	10.04		
	2010	maize	18.06	05.10	05.08	25.06; 05.08		Unpublished
	2011	wheat	10.10	12.06	10.10; 14.04	26.11; 14.04; 23.05		
	2011	maize	18.06	05.10	09.08	20.06; 09.08		
	2012	wheat	10.10	13.06	10.10; 17.04	17.04; 17.05		
	2012	maize	15.06	2.10	03.08	23.06		
	2013	wheat	9.10	14.06	16.10; 13.04;	16.10; 10.12; 13.04		
B	2001	maize	12.06	28.09	05.07; 13.08	05.07; 13.08	80% removed (jointly with grain); 20% left (fallen leaf and stem); 10 cm stubble left.	[9], [10], [11]
	2002	wheat	12.10	08.06	10.10; 15.03	06.10; 30.11; 15.03; 25.04; 30.05		
	2002	maize	15.06	28.09	16.06; 17.07	16.06; 17.07; 20.08		
	2003	wheat	16.10	10.06	16.10; 12.04	07.10; 16.11; 12.04		
	2003	maize	15.06	02.10	15.07; 14.08	23.06; 15.07; 14.08		
	2004	wheat	14.10	08.06	14.10; 22.03	22.03; 12.05		

**Table 3:** Soil water retention and hydraulic parameters used in *Daisy* to simulate maize and winter wheat fields at Luancheng station, the North China Plain.

Soil parameter (symbol, unit)	Soil depth (cm)								Determination^a^
	0–20		20–40		40–110		100–210		
Field	A	B	A	B	A	B	A	B	
Hydraulic conductivity (*Ksat*, cm h^−1^)	1.56	4.65	10.3	2.5	22.3	1.0	0.12	0.1	C
Saturated soil water (*Theta_sat*, vol%)	0.39	0.45	0.44	0.42	0.43	0.40	0.41	0.41	M
Residual soil water (*Theta_res*, vol%)	0.06	0.06	0.01	0.01	0.12	0.05	0.12	0.15	M
van Genuchten α (*alpha*, cm^−1^)	0.008	0.004	0.009	0.004	0.005	0.003	0.002	0.002	C
van Genuchten n (*n*)	1.332	1.252	1.248	1.23	1.186	1.288	1.188	1.25	C
Tortuosity factor (*l*)	1.45	1.75	−1.26	1.23	0.72	1.05	0.88	1.15	C

a M—measured, C—calibrated.

**Table 4:** Crop parameters used in *Daisy* to simulate maize and winter wheat fields at Luancheng station, the North China Plain.

Crop parameter (symbol, unit)	“Pioneer maize”		“Winter wheat”		Determi- nation^a^
	Default	Calibrated	Default	Calibrated	
Soil temperature sum at emergence (*EmrTSum*, °C d)	100	200	100	100	M
Vegetative development rate (*DSRate1*, day^−1^)	0.0245	0.0265	0.026	0.026	S,C
Maximum leaf photosynthesis (*Fm*, g CO_2_ m^−2^h^−1^)	6.0	7.4	5.0	7.0	S,C,L
Quantum efficiency (*QEff*, (g CO_2_ m^−2^h^−1^)/(Wm^−2^))	0.04	0.045	0.05	0.052	S,C,L
Specific leaf weight (*SpLAI*, (m^2^m^−2^)/(g DM m^−2^))	0.03	0.02	0.022	0.0201	M
Conversion efficiencies (*E_Leaf*/*E_Stem*/*E_SOrg*)	0.68/0.66/0.68	0.68/0.60/0.75	0.68/0.66/0.70	0.78/0.78/0.75	S,C
Respiration coefficients *(r_Leaf*/*r_Stem/r_SOrg*)	0.016/0.010/0.010	0.015/0.015/0.005	0.016/0.010/0.010	0.010/0.010/0.005	S,C
Root maximum penetration (*MaxPen*, cm)	120	150	120	210	M
Max NH_4_-N uptake (*MxNH4Up*, g cm^−1^h^−1^)	2.5 × 10^−8^	1.5 × 10^−7^	2.5 × 10^−7^	2.5 × 10^−7^	C,L
Max NO_3_-N uptake (*MxNO3Up*, g cm^−1^h^−1^)	2.5 × 10^−8^	1.5 × 10^−7^	2.5 × 10^−7^	2.5 × 10^−7^	C,L
Specific root length (*SpRtLength*, m g^−1^)	100	130	100	100	M
Potential evapotranspiration factor at DS^b^: 0.0	1.0	1.0	1.0	1.0	L
1.0	1.0	1.3	1.0	1.2	
2.0	1.0	1.2	1.0	1.1	
Leaf weight modifier (*LeafAIMod*) at DS^b^: 0.0	1.0	0.7	1.0	0.8	C
0.5	1.0	0.8	1.0	0.9	
1.0	1.0	1.2	1.0	0.7	
2.0	1.0	0.2	1.0	0.5	
Carbon-nitrogen ratio of slowly-degradable pool of leaf and stem (*C_per_N* in CROP-SLOW)	100	60	100	100	C,L

a M—measured, C—calibrated, S—sensitivity, L—literature.

b DS—development stage: 0—emergence, 0.5—juvenile, 1—flowering, 2—maturity.

**Table 5:** Turnover parameters used in *Daisy* to simulate maize and winter wheat fields at Luancheng station, the North China Plain.

Soil parameter (*symbol*, unit)	Soil depth (cm)					Determi-nation^a^
	0–20		20–40	40–110	100–210	
Organic matter content (*humus*, %)	1.5		0.5	0.5	0.05	
Soil organic matter distribution (*SOM*_*fractions)*	(0.48 0.42 0.10)	(0.42 0.42 0.16)	(0.25 0.25 0.50)	(0.0 0.0 1.0)	(0.0 0.0 1.0)	S,C
Soil carbon-nitrogen ratio (C_*per*_N)	8–12	10–12	12	12		L
Soil microbial biomass carbon-nitrogen ratio (C_*per*_N)	4.8					L
Denitrification factor (*water_factor*)^b^	(0.89 0.01) (0.98 0.01) (1.00 0.01)					C
End of root zone (*MaxRootingDepth*, cm)	190					M
Total carbon added (*Input*,kg C ha^-1^ y^-1^) ^c^	1550 (field A), 800 (field B)					C,L
Root carbon added (*Root*,kg C ha^-1^ y^-1^) ^c^	750 (field A), 600 (field B)					C,L
Depth of non-root input (end, cm)^c^	25					C,L

a M–measured, C–calibrated, S–sensitivity, L–literature.

b Fitting parameter with points (x,y) where first values (x) is fraction of maximal soil water content and second value (y) is denitrification factor.

c Initialisation parameters.

**Table 6:** Model calibration performance for the field experiments at Luancheng station, North China Plain. Values are pooled for the calibration periods of each variable. Values in brackets are one standard deviation of the mean.

Variable	Field	Model	*RMSR*	*Dev*	*ME*	*R^2^*
Maize biomass (Mg ha^−1^)						
Leaf	1.7 (0.3)	1.7 (0.2)	0.37	0.02	0.80	0.81
Stem	2.0 (1.2)	1.8 (0.8)	0.66	−0.29	0.71	0.88
Grain	5.7 (4.2)	5.2 (3.7)	0.77	−0.39	0.96	0.98
Aboveground	7.5 (6.0)	7.0 (5.0)	1.37	−0.48	0.94	0.97
Maize nitrogen (kg N ha^−1^)						
Leaf	34 (16.0)	32 (12.0)	10.09	−2.05	0.70	0.71
Stem	19 (15.0)	18 (10.0)	8.69	−0.74	0.64	0.70
Grain	70 (63.0)	69 (55.0)	12.91	−1.72	0.95	0.97
Aboveground	107 (70.0)	101 (63.0)	19.84	−5.94	0.92	0.93
Winter wheat biomass (Mg ha^−1^)						
Leaf	1.0 (0.5)	1.2 (0.9)	0.86	1.81	0.78	0.91
Stem	2.5 (2.2)	2.3 (2.5)	1.12	−0.82	0.89	0.92
Grain	4.2 (1.4)	4.6 (1.8)	0.71	0.23	0.88	0.94
Aboveground	6.5 (4.6)	6.9 (5.2)	1.03	−0.06	0.98	0.98
Winter wheat nitrogen (kg N ha^−1^)						
Leaf	14 (11.0)	15 (11.0)	5.22	−0.71	0.76	0.77
Stem	25 (28.0)	25 (29.0)	13.88	−0.15	0.57	0.60
Grain	100 (35.0)	99 (34.0)	6.68	−0.73	0.63	0.75
Aboveground	93 (77.0)	93 (79.0)	15.05	−0.96	0.81	0.82
Soil water (vol.%) at depth: 20 cm	35 (4)	33 (3)	4.32	−0.02	0.32	0.65
100 cm	29 (2)	30 (2)	2.67	0.00	0.37	0.30
180 cm	39 (1)	38 (1)	1.91	−0.01	0.72	0.43
Evapotranspiration (mm month^−1^)	77	70	38.21	−8.17	0.68	0.73
Soil water drainage (mm ha^−1^ year^−1^)	55 (11)	50 (18)	9.24	−5.53	0.87	0.80
Nitrate leaching (kg N ha^−1^ year^−1^)	37 (32)	40 (38)	7.85	5.09	0.92	0.97

**Table 7:** Model validation performance for the field experiments at Luancheng station, North China Plain. Values are pooled for the validation periods of each variable. Values in brackets are one standard deviation of the mean.

Variable	Field	Model	*RMSR*	*Dev*	*ME*	*R^2^*
Maize biomass (Mg ha^−1^)						
Grain	7.2 (0.1)	7.5 (0.4)	1.01	0.22	0.35	0.39
Aboveground	11.8 (1.4)	12.7 (0.4)	1.28	0.72	0.64	0.75
Maize nitrogen (kg N ha^−1^)						
Grain	105 (17)	121 (4.7)	22.40	20.04	0.17	0.24
Aboveground	166 (5.8)	182 (2.4)	17.94	16.24	−0.77	0.22
Winter wheat biomass (Mg ha^−1^)						
Grain	6.4 (0.8)	5.6 (0.5)	1.10	−0.68	0.17	0.14
Aboveground	12.1 (1.4)	11.3 (0.8)	1.30	−0.92	0.79	0.38
Winter wheat nitrogen (kg N ha^−1^)						
Grain	130 (11.8)	121 (24.2)	17.27	−8.54	0.18	0.58
Aboveground	155 (18)	175 (33.7)	31.09	20.70	−1.82	0.45
Soil water (vol.%) at: 20 cm	31 (5)	29 (6)	6.44	0.02	−0.17	0.35
100 cm	26 (4)	25 (6)	3.94	−0.01	−0.03	0.76
180 cm	36 (3)	34 (4)	4.75	−0.02	0.01	0.29
Evapotranspiration (mm month^−1^)	83	73	30.34	−10.81	0.75	0.82
Soil water drainage (mm ha^−1^ year^−1^)	51 (14)	40 (4)	16.48	−11.62	0.12	0.10
Nitrate leaching (kg N ha^−1^ year^−1^)	45 (38)	34 (31)	16.68	−8.60	0.81	0.93

**Table 8:** Field and model nitrogen balance components for the experiments at Luancheng station, North China Plain. Values in brackets are one standard deviation of the mean.

Component (kg N ha^−1^ year^−1^)	Field				Model			
	N0	N200	N400	N600	N0	N200	N400	N600
Annual fertilisation	0	200	400	600	0	200	400	600
Crop harvest	78.0 (25)	215.5 (40)	234.0 (29)	242.0 (35)	77.6 (14)	221.2 (28)	252.2 (10)	257.2 (9)
Leaching	1.0 (2)	6.7 (3)	51.7 (9)	88.2 (18)	3.0 (2)	2.9 (4)	37.5 (21)	83.0 (12)
Atmospheric deposition	60–90				62 (18)			
Ammonia volatilisation	0–80				0	10	45	60
N_2_O emission^a^	2.0	3.0	5.0	8.0	3.5	7.0	11.0	13.0
Straw mineralisation	20–120				20 (7)	38 (12)	61 (13)	76 (18)

a Nitrification—born N_2_O.
